# Tourniquet, tranexamic acid, and computer-assisted cryotherapy in primary total knee arthroplasty: A retrospective cohort study

**DOI:** 10.1097/MD.0000000000048909

**Published:** 2026-05-15

**Authors:** Kemal Gokkus, Ahmet Aybar

**Affiliations:** a Baskent University School of Medicine Alanya Research and Practice Center, Antalya, Turkey; b Orthopedics and Trauma Department, University of Health Sciences Gaziosmanpaşa Training and Research Hospital. Karayollari Mahallesi, Istanbul, Turkey.

**Keywords:** blood loss, cryotherapy, postoperative pain, total knee arthroplasty, tourniquet, tranexamic acid

## Abstract

This study aimed to evaluate the comparative efficacy of 4 peri-operative strategies – tourniquet-alone (T-only), tourniquet with computer-assisted cryotherapy (T + CAC), tourniquet with combined intravenous (IV) and intra-articular (IA) tranexamic acid (T + TXA), and a tourniquet-free approach (T-free) – on hemostasis, pain management, and early functional recovery following primary total knee arthroplasty (TKA). In this retrospective comparative study, 213 patients undergoing primary TKA were stratified into 4 groups: Group 1 (tourniquet-only, n = 54), Group 2 (tourniquet + CAC, n = 52), Group 3 (tourniquet + TXA, n = 55), and Group 4 (tourniquet-free, n = 52). Outcome measures included surgical duration, intra-operative and post-operative blood loss, hemoglobin levels (pre-operative and post-operative days 1 and 3), length of stay (LOS), transfusion rates, inflammatory markers (C-reactive protein and erythrocyte sedimentation rate), numerical rating scale (NRS) pain scores, and opioid consumption. The tourniquet + TXA group demonstrated the lowest intra-operative bleeding and post-operative drainage volumes (*P* < .001). Crucially, this protocol resulted in significantly superior hemoglobin preservation on post-operative day 1 (*P* < .001) compared with the tourniquet-only and cryotherapy groups. On post-operative day 3, although the overall variance analysis indicated a trend (*P* = .064), the specific post hoc evaluation confirmed a statistically significant advantage of the tourniquet + TXA protocol. Even after rigorous statistical correction, the TXA group maintained significantly higher hemoglobin levels than the tourniquet-only group (*P* adj. = .050), demonstrating a sustained clinical benefit in blood conservation. The tourniquet + CAC group exhibited the most favorable analgesic profile, with significantly lower NRS pain scores and reduced opioid consumption (*P* = .004). The tourniquet-free group had the longest surgical duration (*P* < .001). The tourniquet-only group demonstrated the highest LOS. A pneumatic tourniquet used alongside combined IV and IA TXA provides the most effective strategy for minimizing blood loss and preserving hemoglobin levels, achieving hemostatic safety comparable to that of tourniquet-free surgery without prolonging operative time. Concurrently, CAC significantly enhanced post-operative analgesia and reduced opioid reliance. These findings suggest that TXA may improve peri-operative blood management, whereas CAC may provide better early analgesic control, thereby helping clinicians tailor peri-operative strategies to enhance recovery and patient safety after TKA.

## 1. Introduction

Total knee arthroplasty (TKA) is a widely performed procedure to alleviate pain and improve function in patients with advanced knee osteoarthritis. Despite its clinical success, TKA presents notable peri-operative challenges including blood loss, post-operative pain, inflammation, and prolonged rehabilitation. Strategies such as pneumatic tourniquets, tranexamic acid (TXA), and cryotherapy are frequently employed to address these issues.

Tourniquet use is traditionally employed to provide a bloodless surgical field, enhance visualization, and reduce the operation time.^[1–3]^ However, its routine application remains controversial due to concerns regarding post-operative pain, delayed recovery, and thromboembolic complications.^[1,2,4,5]^ Meanwhile, TXA has gained widespread use owing to its efficacy in minimizing blood loss and transfusion requirements, with a favorable safety profile even in high-risk populations.^[6–11]^ Similarly, cryotherapy, particularly computer-assisted modalities, has been adopted to reduce pain, swelling, and opioid consumption post-operatively.^[12,13]^

While the individual hemostatic and analgesic efficacies of TXA and computer-assisted cryotherapy (CAC) are well-documented in the literature, previous studies have predominantly focused on dual comparisons or isolated modality assessments.

To our knowledge, there is a paucity of comprehensive comparative studies that simultaneously evaluate the interplay between surgical strategy (tourniquet vs tourniquet-free) and these pharmacological (TXA) and mechanical (CAC) adjuncts within a single cohort. By analyzing a homogeneous cohort of over 200 patients, this study aims to address this gap and to determine whether pharmacological or mechanical adjuncts provide superior outcomes when combined with, or used instead of, tourniquet application.

This study aimed to retrospectively evaluate and compare the clinical and surgical outcomes of 4 distinct peri-operative strategies for TKA: tourniquet alone, tourniquet with cryotherapy, tourniquet with TXA, and tourniquet-free approach. By analyzing surgical duration, blood loss, post-operative inflammation, analgesic requirements, and functional recovery, this study sought to provide a comprehensive assessment to determine the optimal combination for improving peri-operative safety and recovery in TKA.

## 2. Materials and methods

This study was approved by the Gaziosmanpaşa Research and Training Hospital Ethics Committee (May 11, 2022, Protocol ID: 69) and was conducted in accordance with the Declaration of Helsinki. All participants signed informed consent forms prior to study inclusion.

In this retrospective cohort study, the medical records of 246 consecutive patients who underwent unilateral primary TKA between December 2014, and April 2022, were reviewed. Patients were excluded if they had contraindications to TXA or cold intolerance; were outside the age range of 60 to 79 years; or had medical conditions impairing vascular, hepatic, renal, cardiopulmonary, rheumatologic, hematologic, or cognitive function.

Additional exclusion criteria included prior ipsilateral knee surgery, active psychiatric illness, coagulopathy, pre-operative anemia, active thromboembolic disease, or inability to comply with post-operative rehabilitation or follow-up.

After applying these criteria, 33 patients were excluded and 213 met all eligibility requirements, forming the final analytic cohort. Patients were grouped according to the peri-operative strategy employed by the attending surgeon, with comparable baseline demographics across groups, although retrospective allocation may have introduced selection bias.

Patients were allocated into 4 groups: Group 1, tourniquet alone (T-only; tourniquet: VBM 2800 TM, Netherlands); Group 2, tourniquet plus computer-assisted cryotherapy (T + CAC); Group 3, tourniquet plus tranexamic acid (T + TXA); and Group 4, tourniquet-free (T-free).

All surgeries were performed by 2 experienced orthopedic surgeons using a standardized medial parapatellar approach with preservation of the posterior cruciate ligament, and the same type of prosthesis was implanted in all cases. Two grams of intravenous (IV) cefazolin was administered prior to tourniquet inflation.

**Figure 1.**
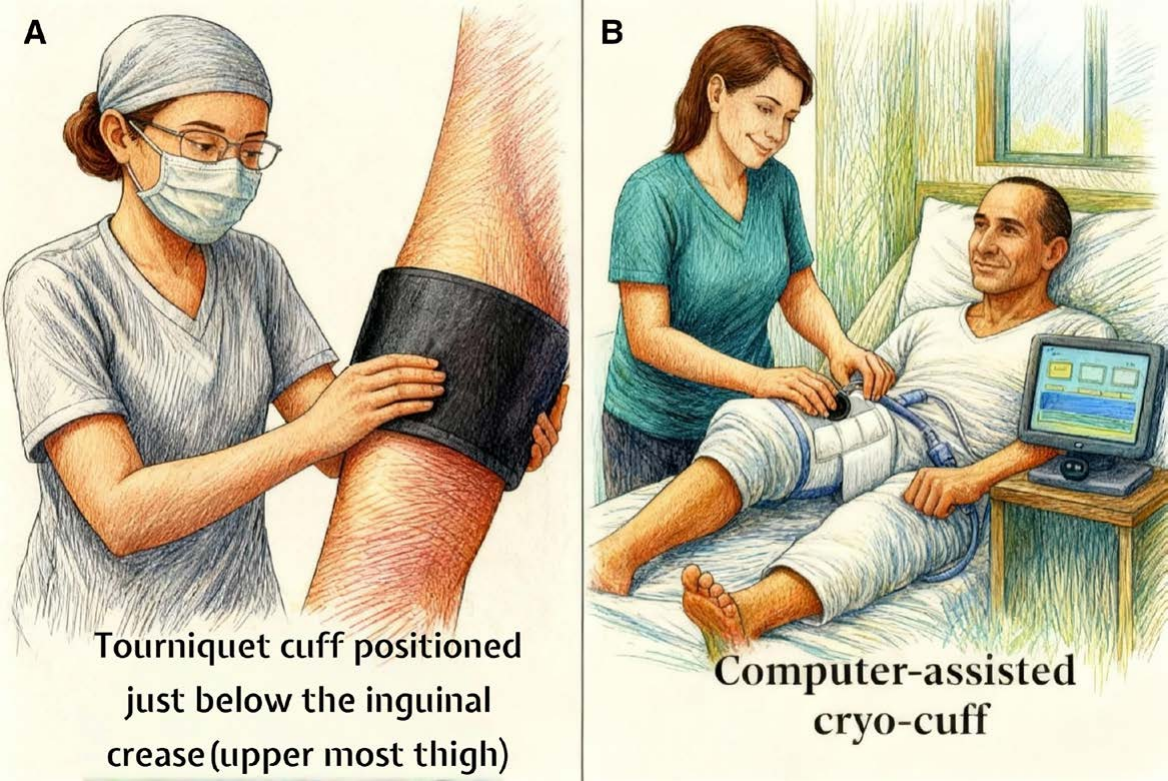
Illustration of the peri-operative interventions: (A) Placement of the pneumatic tourniquet cuff positioned just below the inguinal crease. (B) Application of the computer-assisted cryotherapy cuff (Cryo/Cuff) for post-operative care.

A pneumatic tourniquet cuff sized to the thigh circumference was positioned in all cases (Fig. 1A); when indicated, it was inflated to 100 mm Hg above the patient’s systolic blood pressure immediately before skin incision and deflated after cementation was completed.

Meticulous hemostasis was ensured, no compressive bandage was used, and post-operative drainage was through a single deep intra-articular (IA) closed-suction drain, typically removed within 24 to 48 hours. From the pre-operative period (on the day of hospitalization before cryotherapy started) through post-operative day 3, patients’ pain levels were assessed at the bedside using the numerical rating scale (NRS). The analgesic protocol followed a stepwise procedure. Initially, patients reporting pain received 1 g of IV paracetamol (100 mL; paracetamol 10 mg/mL infusion solution) administered over 15 to 30 minutes. If the patient reported no relief after infusion, diclofenac sodium (75 mg/3 mL) was administered intramuscularly. In cases in which pain persisted despite this dual regimen and the NRS score remained >7, pethidine hydrochloride (100 mg/2 mL) was administered intramuscularly. From the pre-operative and post-operative last visit (mean 8^**th**^ month), Hospital for Special Surgery (HSS) scores were assessed in the outpatient clinic by experienced residents.

### 2.1. Group 1 – tourniquet group (T-only)

The procedure was performed following the standard tourniquet protocol described above without any additional peri-operative interventions (Fig. 1A).

### 2.2. Group 2 – tourniquet + computer-assisted cryotherapy group (T + CAC)

In addition to the standard tourniquet protocol, the Cryo/Cuff-Waegener™ device circulated chilled water through the knee cuff under regulated pressure. Cooling sessions were initiated 2 hours pre-operatively (reservoir temperature: 8–10°C; at least 2 hours before surgery), followed by a 3-hour post-operative session immediately after surgery (reservoir temperature: 8–10°C). On post-operative day 1, 2 sessions of 2 hours each were applied (reservoir temperature: 13–15°C), and on thefollowing4 days, 3 sessions of 1 hour each were continued daily at the same temperature range (13–15°C) (Fig. 1B). This protocol was adapted from Kuyucu et al,^[14]^ who previously reported favorable clinical outcomes.

### 2.3. Group 3 – tourniquet + tranexamic acid (TXA) group (T + TXA)

Intravenous TXA (15 mg/kg in 100 mL isotonic solution) was infused 10 to 15 minutes prior to tourniquet inflation. Following tourniquet deflation at the end of the surgery, an additional 1 gram of TXA was applied intra-articularly.

### 2.4. Group 4 – tourniquet-free (T-free)

No tourniquet cuff was applied, and other routine procedures were performed as described above. Allogenic blood transfusions were managed using a symptom-guided restrictive strategy to prioritize patient safety in this elderly cohort. Transfusion was considered for patients with hemoglobin levels between 7 to 9 g/dL if accompanied by clinical signs of anemia (e.g., tachycardia, hypotension, dizziness) or significant cardiovascular comorbidities, rather than relying solely on a strict restrictive numerical trigger.

The baseline demographics (age, sex, body mass index, pre-operative hemoglobin) values have been documented (Table 1).

**Table 1:** Baseline characteristics of the study population.

Variable	T-only (n = 54)	T + CAC (n = 52)	T + TXA (n = 55)	T-free (n = 52)	*P*-value†
Age (years), mean ± SD	69.2 ± 4.7	69.5 ± 5.0	69.8 ± 4.8	69.5 ± 4.9	>.05
Sex (Male/ Female)	6/ 48	9/ 43	9/ 46	12/ 40	>.05
BMI (kg/m^2^), mean ± SD	34.1 ± 2.3	34.0 ± 2.2	34.2 ± 2.1	33.9 ± 2.4	>.05
Pre-operative Hb (g/dL)	13.1 ± 0.9	13.0 ± 0.8	13.2 ± 0.9	13.1 ± 0.8	>.05

BMI = body mass index, Hb = hemoglobin, SD = standard deviation, T + CAC = tourniquet with computer assisted cryotherapy, T-free = tourniquet free, T-only = with only tourniquet, T + TXA = tourniquet with combined intravenous and intra-articular tranexamic acid.

* *P*-values derived from Kruskal–Wallis (for continuous variables) or Chi-square test (for categorical variables). No statistically significant differences were observed among groups at baseline.

## 3. Statistical method

All statistical analyses were performed using IBM SPSS Statistics for Windows, Version 25 (IBM Corp., Armonk). Data normality was assessed using the Kolmogorov–Smirnov test and visual inspection. Given the non-normal distribution of most variables, specifically the post-operative floor effects and skewness observed in hemoglobin and inflammatory markers, nonparametric statistical methods were applied. Continuous variables were compared among the 4 groups using the Kruskal–Wallis test, followed by Dunn’s post hoc pairwise analysis with Bonferroni correction.

Categorical variables were analyzed using the Pearson chi-square test with Bonferroni-adjusted *Z*-tests for multiple comparisons. The Fisher–Freeman–Halton exact test was used for complication analysis due to the low number of events and expected cell counts <5.

Transfusion outcomes were analyzed in 2 complementary ways. First, the proportion of patients who received at least one allogeneic red blood cell transfusion in each group was compared using Pearson χ^2^ test. Second, the number of transfused units per patient was compared among the groups using the Kruskal–Wallis test. As an exploratory sensitivity analysis, a Poisson regression model for transfusion counts was also fitted; the global model p-values are reported in Table 2.

**Table 2:** Transfusion requirements among groups.

Variable	T-only (n = 54)	T + CAC (n = 52)	T + TXA (n = 55)	T-free (n = 52)	*P*-value
Patients transfused, n (%)	21 (38.9%)	16 (30.8%)	14 (25.5%)	21 (40.4%)	.313*
Total units administered, n	26	24	19	29	.440†

T + CAC = tourniquet with computer assisted cryotherapy, T-free = tourniquet free, T-only = with only tourniquet, T + TXA = tourniquet with combined intravenous and intra-articular tranexamic acid.

* Pearson Chi-square test.

† Kruskal–Wallis test comparing total units per patient.

Pain intensity was measured using a 10-point NRS (0 = no pain, 10 = worst imaginable pain) pre-operatively and on post-operative days 1 and 3. NRS scores at each time point were compared among the 4 groups using the same nonparametric strategy (Kruskal–Wallis with Dunn post hoc tests and Bonferroni correction). Post hoc power analysis indicated adequate statistical power (0.91–1.00) for detecting clinically meaningful differences across primary outcomes. Parameters such as intra-operative bleeding, drain output, surgery duration, and length of stay (LOS) demonstrated large effect sizes (η^2^ ≥ 0.14), supporting the study’s ability to detect clinically meaningful differences.

C-reactive protein (CRP) and erythrocyte sedimentation rate (ESR) showed moderate-to-large effect sizes, reflecting meaningful yet subtle distinctions in post-operative inflammation. With a total sample size of n = 213 distributed across 4 balanced groups, the study achieved excellent sensitivity to detect between-group differences even under nonparametric conditions. Baseline demographic data are presented as mean ± standard deviation, while non-normally distributed clinical outcomes are presented as median with interquartile range [25th–75th percentiles]. *P* < .05 was considered statistically significant.

## 4. Results

### 4.1. Baseline demographics

The baseline demographic characteristics, including age, sex distribution, and body mass index, were comparable across all groups with no statistically significant differences (*P* > .05). Similarly, pre-operative hemoglobin levels showed no significant variation among the study cohorts (*P* > .05). The detailed baseline characteristics of the study population are summarized in Table 1.

### 4.2. Surgical duration and blood loss

Median operative duration was significantly longer in the tourniquet-free group compared with the other 3 groups (87.0 [82.0–90.0] min vs 72.0–75.5 min, *H* = 92.3, *P* < .001). Pairwise comparisons confirmed that tourniquet use significantly shortened operative time (*P* < .001). Intra-operative blood loss was significantly lower in the T + TXA group than in the tourniquet-free and tourniquet-only groups (*P* < .001). Although the T-free group exhibited the greatest intra-operative bleeding, it did not demonstrate a proportionally increased post-operative drainage (Table 3).

**Table 3:** Perioperative and early post-operative outcomes (median [IQR]).

Outcome measure	T-only (n = 54)	T + CAC (n = 52)	T + TXA (n = 55)	T-free (n = 52)	*P*-value*
Surgical duration (min)	75.5 [72.0–80.0]	74.0 [70.0–78.0]	72.0 [70.0–76.0]	87.0 [82.0–90.0]	**<.001**
Intra-operative bleeding (mL)	280 [250–300]	225 [200–270]	200 [180–215]	335 [300–380]	**<.001**
Drain output (mL)	410 [400–450]	380 [340–410]	300 [270–355]	350 [305–400]	**<.001**
LOS (d)	9.0 [8.0–9.0]	7.0 [7.0–8.0]	7.0 [6.0–8.0]	7.0 [7.0–8.0]	**<.001**
Hb day 1 (g/dL)	**10.0** [9.4–10.8]	**10.4** [9.8–11.2]	**11.2** [10.6–12.2]	10.1 [9.4–11.0]	<.001
Hb day 3 (g/dL)	**9.2** [8.4–9.9]	**10.0** [9.4–10.8]	**10.4** [9.8–11.5]	**9.6** [8.8–10.4]	.064*
CRP day 1 (mg/L)	53.5 [42.0–62.6]	48.0 [40.0–58.5]	60.0 [55.0–66.0]	54.5 [46.2–61.5]	**<.001**
ESR day 1 (mm/h)	38.0 [33.0–44.0]	24.0 [18.8–45.8]	30.0 [22.0–40.0]	42.0 [34.8–46.2]	**<.001**
Paracetamol (vials)	3.0 [3.0–4.0]	**2.0 [2.0–3.0]**	3.0 [3.0–4.0]	3.0 [3.0–4.0]	**.014** †
Diclofenac (mg)	375 [300–375]	300 [300–375]	375 [300–375]	337 [300–375]	**<.001**
Pethidine (mg)	0 [0–100]	0 [0–0]	0 [0–50]	0 [0–0]	**.004**

CRP = C-reactive protein, ESR = erythrocyte sedimentation rate, Hb = hemoglobin, IQR = interquartile range T + CAC = tourniquet with computer assisted cryotherapy, T-free = tourniquet free, T-only = with only tourniquet, T + TXA = tourniquet with combined intravenous and intra-articular tranexamic acid.

* *P*-values were calculated using the Kruskal–Wallis test. Bold values indicate statistical significance (*P* < .05). post hoc pairwise comparisons (Dunn–Bonferroni) demonstrated significantly higher hemoglobin levels in the tourniquet + TXA group compared with the tourniquet-only group on postoperative day 1. On postoperative day 3, although the overall Kruskal–Wallis test indicated a non-significant trend, post hoc Dunn–Bonferroni analysis revealed a statistically significant difference between the 2 groups (*P* adj = .050).

† Although median values appear identical, the statistically significant difference reflects variations in rank distribution (mean ranks) among groups.

### 4.3. Post-operative drainage and hemoglobin

**Figure 2.**
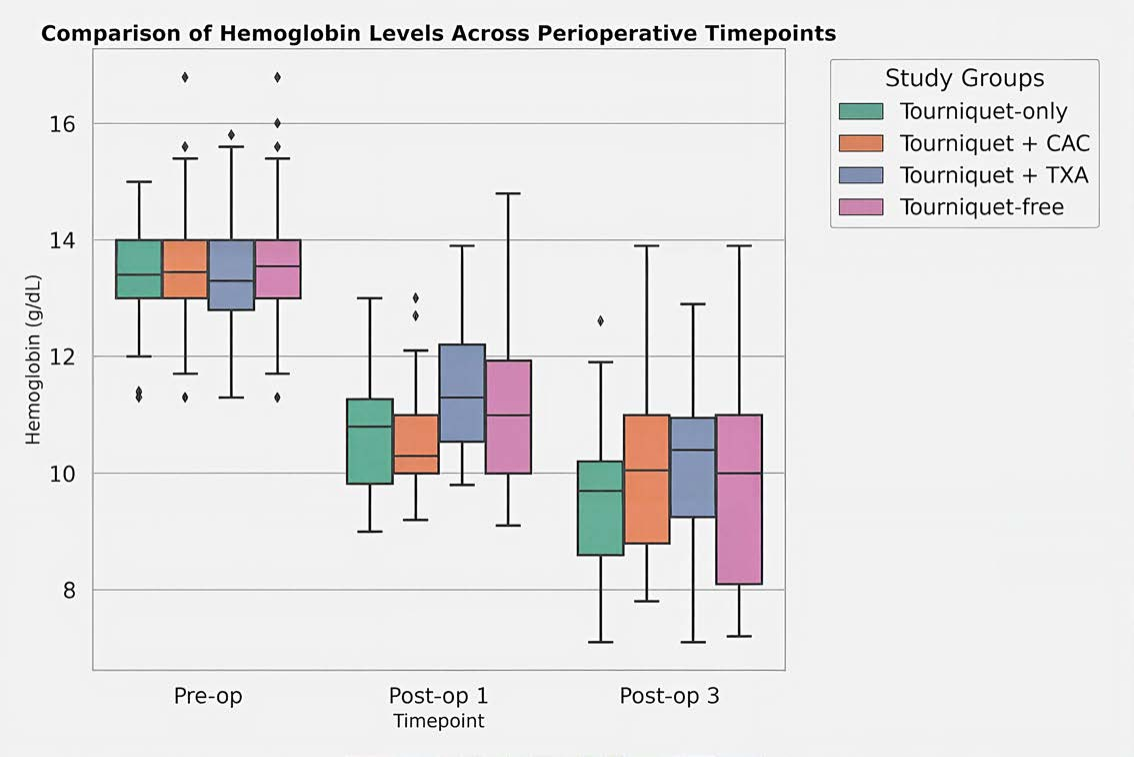
Box plot comparison of hemoglobin levels (g/dL) across the 4 study groups at pre-operative, post-operative day 1, and post-operative day-3 time points. The boxes represent the interquartile range (IQR), the horizontal line within the box indicates the median, and the whiskers extend to the minimum and maximum values (excluding outliers). Key findings: Pre-operative: No significant differences were observed among groups (*P* > .05). Post-operative day 1: The tourniquet + TXA group (green) demonstrated significantly higher median hemoglobin levels compared to the tourniquet-only and tourniquet + CAC groups (*P* < .001). Post-operative day 3: The tourniquet + TXA group maintained the highest median hemoglobin levels, showing a sustained numerical advantage and statistical superiority over the tourniquet-only group (*P* adj = .050), comparable to the tourniquet-free approach. CAC = computer-assisted cryotherapy, TXA = tranexamic acid, Adj = adjusted.

Median drain output was highest in the T-only group (410 [400–450] mL) and lowest in the T + TXA group (300 [270–355] mL), with significant differences among groups (*H* = 54.4, *P* < .001). All 3 alternative strategies demonstrated significantly lower drainage rates than the T-only group (*P* < .01). The baseline demographic characteristics and pre-operative hemoglobin levels were comparable among the 4 groups (*P* > .05). Owing to non-normal data distribution, post-operative hemoglobin levels were analyzed using nonparametric methods and are presented as median values with interquartile ranges (Fig. 2). On post-operative day 1, a significant difference in median hemoglobin levels was observed among the groups (Kruskal–Wallis *H* = 16.99, *P* < .001) (Fig. 2). post hoc Dunn–Bonferroni analysis demonstrated that the tourniquet + TXA group maintained significantly higher median hemoglobin levels than both the tourniquet-only group (*P* adj = .010) and the tourniquet + CAC group (*P* adj < .001). On post-operative day 3, the overall group differences demonstrated a non-significant trend (*P* = .064). However, post hoc analysis confirmed that the tourniquet + TXA group maintained significantly higher median hemoglobin levels than the tourniquet-only group (*P* adj = .050), indicating a sustained advantage in hemoglobin preservation (Fig. 2).

### 4.4. Transfusion rates

Transfusion rates were lowest in the T + TXA group (25.5%) and highest in the tourniquet-free (40.4%) and tourniquet-only (38.9%) groups without a statistically significant difference between groups (*P* = .313). Similarly, the total number of transfused units did not differ significantly between groups (*P* = .440). Transfused patients exhibited significantly lower post-operative hemoglobin levels than non-transfused patients (*P* < .001), consistent with the application of a restrictive/symptom-guided transfusion strategy (Table 2).

### 4.5. Inflammatory response (CRP and ESR)

The T + CAC group demonstrated significantly lower CRP and ESR levels on post-operative day 1 compared to all other groups (*P* < .05). Pairwise comparisons among the remaining 3 protocols (T + TXA, T-only, T-free) revealed no statistically significant differences (Table 3).

### 4.6. Pain scores and analgesic use

**Figure 3.**
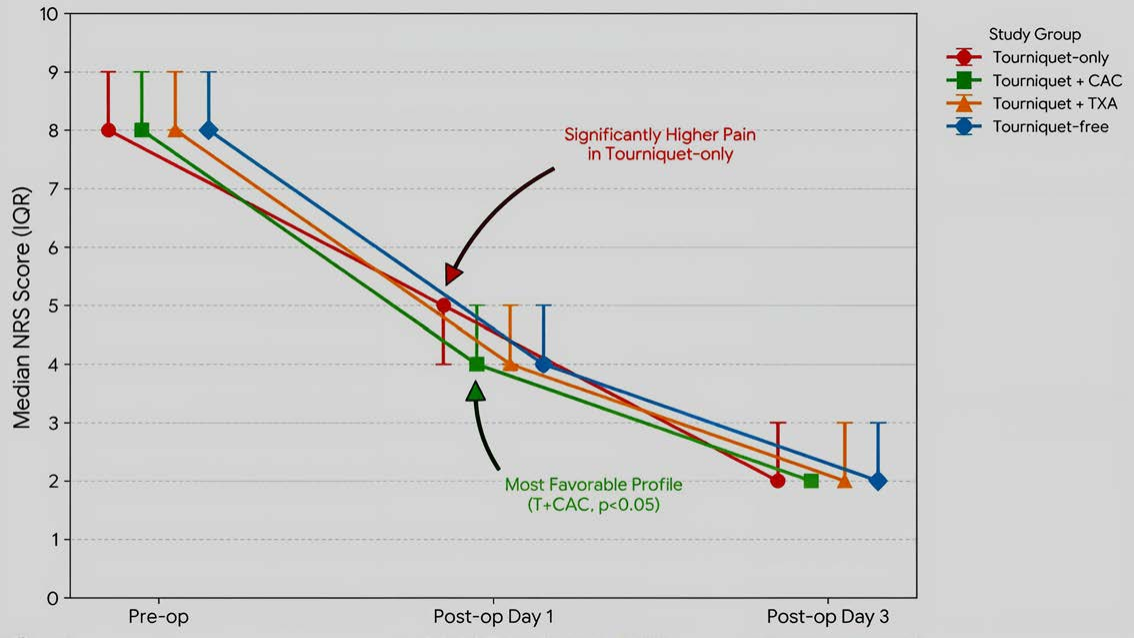
Evolution of post-operative pain scores assessed by the numeric rating scale (NRS). Data are expressed as median and IQR. While baseline scores were comparable, significant intergroup differences were observed on day 1 (*P* = .004), with the T + CAC group demonstrating superior pain relief. By day 3, pain scores converged, and no significant differences remained (*P* > .05). IQR = interquartile range.

In the current study, the T + CAC group demonstrated significantly lower NRS scores on post-operative day 1 than all other groups (Kruskal–Wallis *H* = 13.31, *P* = .004), confirming the early analgesic advantage associated with controlled cryotherapy. Dunn post hoc analysis revealed that the T-only group had significantly higher pain levels than the T + CAC, T + TXA and T-free groups (*P* < .05). The T + CAC therapy group had the lowest NRS values at this point. Pre-operative NRS values were comparable across the groups, indicating that the observed post-operative benefit reflects a true therapeutic effect rather than a baseline analgesic bias. By post-operative day 3, pain scores had decreased substantially in all groups, and no significant inter-group differences were detected in post hoc testing (*P* > .05), suggesting convergence of pain outcomes. Nevertheless, the T + CAC group continued to require the lowest amount of analgesic medication, supporting its role in reducing early opioid and non-opioid exposures (Fig. 3). Analgesic consumption differed significantly among groups, with the T + CAC group demonstrating the lowest paracetamol, diclofenac, and rescue pethidine use, consistent with an analgesic-sparing effect (Table 3).

### 4.7. Functional outcomes and hospitalization

**Figure 4.**
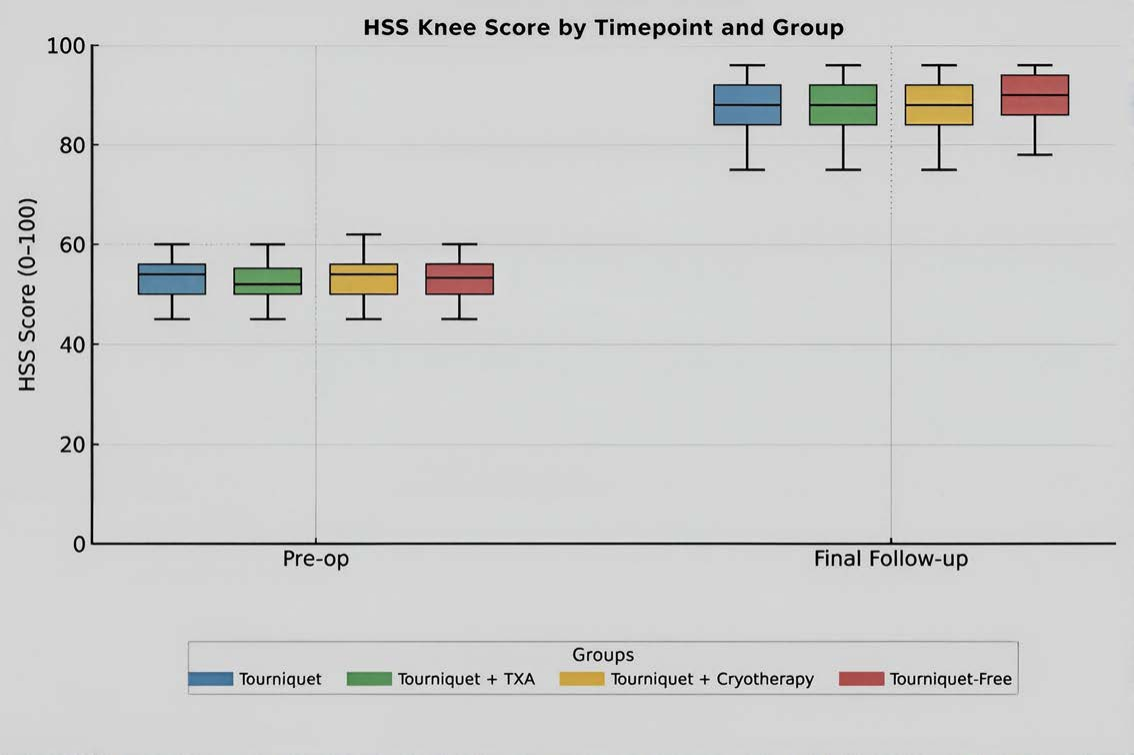
Functional outcomes evaluated using the Hospital for Special Surgery (HSS) knee score. Boxplots represent median values and interquartile ranges at pre-operative baseline and final follow-up (8th month). No statistically significant differences in functional recovery were found among the peri-operative strategies (*P* > .05).

No significant differences were observed in the range of motion or HSS scores at the 8-month follow-up (*P* > .05) (Fig. 4). LOS differed significantly among the groups (*H* = 67.1, *P* < .001). It was longest in the T-only cohort (9.0 [8.0–9.0] days), whereas patients in the T + TXA (7.0 [6.0–8.0] days), T + CAC (7.0 [7.0–8.0] days), and T-free (7.0 [7.0–8.0] days) groups were discharged earlier.

### 4.8. Complications

The overall complication rate was 3.8% (8 events among 213 patients). The complication profile included superficial/supra-fascial wound infections (n = 6), joint hematoma (n = 1), and one inpatient fall (n = 1). No deep periprosthetic infections, deep vein thrombosis (DVT), or pulmonary embolism were observed in any group (Table 4).

**Table 4:** Post-operative complication profile by perioperative strategy group.

Complication	T-only (n = 54)	T + CAC (n = 52)	T + TXA (n = 55)	T-free (n = 52)	*P*-value
Superficial/ Supra-fascial infection, n (%)	2 (3.7%)	1 (1.9%)	1 (1.8%)	2 (3.8%)	N/A†
Joint hematoma, n (%)	1 (1.9%)	0 (0.0%)	0 (0.0%)	0 (0.0%)	N/A†
Fall, n (%)	0 (0.0%)	0 (0.0%)	0 (0.0%)	1 (1.9%)	N/A†
Deep vein thrombosis/ Pulmonary embolism, n (%)	0 (0.0%)	0 (0.0%)	0 (0.0%)	0 (0.0%)	N/A†
Skin complication (cryotherapy-related), n (%)	N/A	0 (0.0%)	N/A	N/A	N/A†
**Total complications, n (%**)	**3 (5.6%**)	**1 (1.9%**)	**1 (1.8%**)	**3 (5.8%**)	**.586** *

Values are presented as n (%).

Total complication events (n = 8) were derived from complete retrospective review of the study cohort.

T + CAC = tourniquet with computer assisted cryotherapy, T-free = tourniquet free, T-only = with only tourniquet, T + TXA = tourniquet with combined intravenous and intra-articular tranexamic acid.

* *P* values were calculated using the Fisher–Freeman–Halton exact test due to the low number of events and expected cell counts <5.

† Statistical comparison was not applicable due to insufficient event counts.

Given the very low number of individual events, statistical comparisons were performed only for the overall complication rate rather than for each specific complication. The distribution of total complications across the 4 groups did not differ significantly (Fisher–Freeman–Halton exact test, *P* = .586). Cryotherapy-related skin complications were not observed in the T + CAC group. Detailed complication data are presented in Table 4.

## 5. Discussion

This study evaluated several peri-operative strategies that may influence outcomes after TKA. Although numerous studies have examined the effects of TXA, cryotherapy, and tourniquet use individually, to our knowledge, few studies have directly compared 4 peri-operative approaches within a single cohort.

TXA is a well-established and safe antifibrinolytic agent that effectively reduces peri-operative bleeding after TKA. Evidence from meta-analyses, comprehensive reviews, and large-scale clinical studies has confirmed that TXA does not increase thromboembolic or renal complications,^[6–11]^ while also reducing intraoperative and post-operative blood loss, transfusion rates, and LOS.^[15–21]^

In our study, the TXA-treated group, particularly when combined with tourniquet use demonstrated significantly lower intra-operative bleeding and post-operative drainage, consistent with prior reports.^[22–24]^ Furthermore, this protocol resulted in significantly superior hemoglobin preservation on post-operative days 1 and 3 compared with the other groups.

Despite these hemostatic benefits, no significant differences were observed between the groups in terms of allogeneic blood transfusion rates. Although the tourniquet + TXA group maintained significantly higher hemoglobin levels on post-operative days 1 and 3, this did not result in lower transfusion rates. This finding aligns with those of contemporary restrictive transfusions strategies that utilize a specific physiological threshold (typically 7–8 g/dL) rather than reacting to linear decreases in hemoglobin.^[25]^ From a statistical perspective, hemoglobin is analyzed as a continuous variable, whereas transfusion is a categorical outcome; therefore, statistically significant differences in hemoglobin levels may not necessarily translate into differences in transfusion rates.

Various TXA administration routes, including IV, IA, topical, and oral, have proven effective, with IV and IA being the most commonly used routes.^[26–36]^ In our protocol, IV TXA (15 mg/kg) was administered prior to tourniquet inflation, followed by 1 gram of IA TXA after tourniquet deflation, a regimen supported by previous studies as both safe and effective.^[21,23,33]^ TXA also remains beneficial in tourniquet-free TKA, significantly reducing the hemoglobin drop and transfusion.^[37–39]^ These findings reinforce TXA’s role as a versatile adjunct across surgical techniques, providing reliable blood-sparing benefits without increasing complication risks.

A retrospective study involving 558 TKA patients reported that the combination of TXA and continuous tourniquet use yielded the lowest hemoglobin decline and transfusion rates,^[24]^ whereas limited tourniquet application during cementation produced superior outcomes compared with tourniquet-only surgery.^[40]^ Although our study did not include a TXA + tourniquet-free subgroup, our results similarly emphasize TXA’s potential to optimize blood management when used in conjunction with tourniquet use.

Tourniquet application in TKA remains controversial; although tourniquet use can reduce intraoperative bleeding and improve visualization, total blood loss and transfusion rates often do not differ significantly.^[1,41,42]^ Moreover, tourniquet use may increase post-operative pain, delay recovery, and elevate the risk of distal DVT.^[4,43,44]^ Excessive pressures (>240 mm Hg) may also lead to transient sensory impairment.^[45]^ Some studies reported no long-term functional differences,^[46]^ whereas others noted faster recovery in tourniquet-free TKA.^[4]^

Consequently, selective or limited tourniquet use during cementation combined with TXA has been proposed as a balanced, patient-centered approach.^[47,48]^ Peri-operative management of pain, inflammation, and blood loss remains a central focus in optimizing recovery after TKA. The present investigation, incorporating 4 distinct peri-operative combinations, provided a comprehensive evaluation of hemostatic, inflammatory, analgesic, and functional outcomes that extends beyond the scope of prior reports. Cryotherapy has long been used as an adjunct to reduce post-operative pain, edema, and opioid consumption,^[49]^ and several studies suggest that its combination with TXA may have synergistic benefits without increasing thromboembolic risk.^[50]^ Huang et al^[50]^ reported that the combination of cryotherapy and IA TXA reduced early post-operative pain and peri-operative blood loss following TKA. However, their protocol did not include a tourniquet-free group and did not include detailed analyses of inflammatory markers therefore limiting direct assessment of strategies that avoid tourniquet-related effects. Given the growing interest in tourniquet-free approaches to potentially enhance early functional recovery and minimize soft tissue complications.^[4,41,42]^ Inclusion of this group allows a more balanced and clinically relevant comparison. By incorporating this additional arm into our design, we observed that while the tourniquet + TXA protocol achieved greater hemostatic control, the tourniquet + cryotherapy protocol was associated with improved early analgesic outcomes.

Existing evidence suggests that cryotherapy provides modest but consistent analgesic benefits, with a meta-analysis reporting reductions in post-operative pain and opioid use.^[51]^ However, its superiority over traditional ice packs remains controversial, with some analysis and reports showing minimal differences.^[51,52]^

CAC has shown promising results, particularly in reducing early post-operative edema and pain, through controlled temperature regulation and extended cooling periods.^[12,13]^ Nonetheless, authoritative guidelines by the American Academy of Orthopaedic Surgeons and recent reviews by Thacoor and Sandiford offer only moderate recommendations for routine use, citing variability in protocols and limited comparative evidence.^[53,54]^

Despite the demonstrated effectiveness of standard pain protocols in orthopedic surgery, research specifically addressing peri-operative pain control in patients with high opioid tolerance remains limited.^[55]^ In the current study, the T + CAC group demonstrated significantly lower NRS scores on post-operative day 1 than the other groups. By post-operative day 3, pain levels had markedly decreased in all cohorts, and inter-group differences were no longer statistically significant, although numerical trends continued to favor the CAC strategy. Importantly, although cooling was initiated 2 hours pre-operatively, baseline NRS values were comparable across the groups, indicating that the observed benefit reflects a true therapeutic effect rather than baseline bias. The analgesic consumption findings further reinforced the efficacy of CAC. The T + CAC cohort required significantly fewer paracetamol vials, lower cumulative diclofenac sodium doses, and the least opioid rescue therapy. The substantial inter-group difference in diclofenac usage—highly significant in this analysis–suggests that cryotherapy may meaningfully reduce post-operative soft-tissue inflammation, thereby decreasing reliance on non-steroidal anti-inflammatory drugs. Similarly, the reduced need for opioid rescue underscores the relevance of CAC in minimizing opioid exposure, which is an increasingly important consideration in modern peri-operative care. Taken together, these findings support the integration of CAC into multimodal peri-operative protocols to enhance analgesic outcomes and reduce medication requirements.

Further prospective randomized studies are warranted to validate these findings, optimize timing, and evaluate long-term benefits. Functional outcomes, including range of motion and HSS scores, did not differ significantly among the 4 peri-operative strategies.

The T + CAC strategy yielded distinct benefits in terms of early pain control and reduced analgesic requirements, indicating enhanced patient comfort during the acute post-operative phase. Although tourniquet-only procedures facilitate improved intra-operative visualization and technical ease for the surgeon, these advantages appear counterbalanced by higher post-operative pain levels and prolonged hospitalization, making this approach less favorable from a recovery perspective.

Finally, T-free surgery resulted in greater intra-operative bleeding and higher early inflammatory marker levels than the cryotherapy-assisted protocol, although the post-operative drainage volumes were lower than those in the tourniquet-only and cryotherapy groups. CRP and interleukin-6 are widely used as biomarkers for evaluating post-operative inflammatory responses and surgical stress.^[56]^

The elevated post-operative inflammatory markers (CRP and ESR) in the tourniquet-free group likely reflect greater intra-operative bleeding and increased soft-tissue manipulation required to maintain hemostasis.^[57]^ Conversely, the combination of tourniquet use with cryotherapy yielded the lowest CRP and ESR values, significantly lower than TXA supplementation on the first post-operative day, suggesting that local hypothermia mitigates ischemia-reperfusion-related inflammation. This aligns with the physiological rationale of cold therapy, which reduces tissue edema and inflammatory cytokine activity. Although TXA possesses anti-inflammatory properties by inhibiting fibrinolytic activation,^[58]^ its systemic effect appears insufficient to fully counteract the local ischemia-reperfusion injury caused by the tourniquet, as reflected by the slightly higher CRP values despite effective hemostasis. These findings suggest that cryotherapy may be particularly effective in attenuating the acute inflammatory response.

Although TXA^[6–11,15–22,26–33]^ tourniquet^[1–5,41–46]^ protocols, and cryotherapy^[12,13,49–52]^ have been widely studied in TKA, most previous investigations have evaluated these modalities in isolation or limited their primary outcomes to blood loss or pain alone. In contrast, the present study offers a multidimensional comparison of tourniquet-only, tourniquet + TXA, tourniquet + CAC, and tourniquet-free TKA performed by the same surgical team under uniform peri-operative conditions. This design allowed the differential physiological effects of each strategy to be distinguished more clearly. Our findings demonstrate that TXA provides the most favorable hemostatic profile, whereas CAC optimizes early pain control and inflammatory suppression, and tourniquet-only procedures show the least balanced outcomes. Our findings may help inform peri-operative decision-making and contribute to optimizing patient safety in TKA, a procedure frequently performed in the elderly population at high risk of complications.

The blood-conserving effect of TXA may contribute to improved patient safety by reducing exposure to allogeneic transfusions, thereby reducing the risks associated with transfusion reactions, disease transmission, and immune modulation. Furthermore, the substantial reduction in opioid consumption achieved by the CAC protocol is a vital safety outcome in the current context of the opioid crisis. By providing effective non-pharmacological pain relief, this protocol aids in safer, faster rehabilitation and decreases patient exposure to high-risk analgesics, which is crucial for reducing delirium and dependency in the elderly cohort.

The complication profile observed in the present study further supports the safety of the evaluated peri-operative strategies. The overall complication rate was low (3.8%), which is consistent with contemporary TKA literature. Importantly, no thromboembolic events (DVT or pulmonary embolism) were observed in any group, including the T + TXA cohort. This finding aligns with growing evidence demonstrating that TXA does not increase thromboembolic risk following TKA.

Similarly, no cryotherapy-related skin complications were identified in the T + CAC group, indicating that CAC can be safely implemented when regulated temperature protocols are followed. The absence of significant differences in complication rates among the groups suggests that the peri-operative strategies evaluated in this study can be selected based on their specific clinical benefits without compromising patient safety.

## 6. Conclusion

This study suggests that perioperative strategy selection may meaningfully influence hemostasis, inflammation, and early pain control following TKA. Combined IV and IA TXA, particularly when used with tourniquet application, was associated with reduced intraoperative blood loss, lower postoperative drainage, and improved early hemoglobin preservation, whereas the tourniquet plus CAC protocol demonstrated superior early analgesic outcomes, with lower pain scores, reduced opioid and non-steroidalanti-inflammatory drug consumption, and attenuation of the acute inflammatory response. Taken together, these findings indicate that TXA is associated with improved perioperative blood conservation, whereas CAC is associated with lower early pain scores and reduced analgesic requirements. These modality-specific effects may assist clinicians in selecting and individualizing perioperative strategies to optimize recovery following TKA.

## 7. Limitations

This study has several limitations. First, its retrospective design introduces the potential for selection bias and limits causal inference. Although baseline characteristics were comparable among groups, the absence of randomization and blinding may have allowed unmeasured confounders to influence treatment allocation and outcomes. Second, the single-center setting and operations performed by only 2 surgeons may restrict external validity. Third, long-term functional outcomes and late complications were not systematically evaluated, precluding assessment of sustained benefits or delayed adverse events. Finally, the predominance of female patients may limit generalizability to broader TKA populations. Prospective randomized trials are warranted to confirm these findings and further clarify optimal peri-operative strategies.

## Author contributions

**Conceptualization:** Kemal Gokkus.

**Data curation:** Ahmet Aybar.

**Formal analysis:** Ahmet Aybar.

**Investigation:** Ahmet Aybar.

**Methodology:** Kemal Gokkus, Ahmet Aybar.

**Software:** Kemal Gokkus.

**Writing – original draft:** Kemal Gokkus.

**Writing – review & editing:** Kemal Gokkus, Ahmet Aybar.
